# COVID-19 pandemic and the exchange rate movements: evidence from six major COVID-19 hot spots

**DOI:** 10.1186/s43093-022-00126-8

**Published:** 2022-06-25

**Authors:** Aamir Jamal, Mudaser Ahad Bhat

**Affiliations:** 1 Cluster University-ASC, Srinagar, India; 2 University of Kashmir, Srinagar, India

**Keywords:** COVID-19, Exchange rate, Asset-market approach, Panel ARDL, F400, F310

## Abstract

The study’s primary objective is to unravel the nexus between the COVID-19 crisis and the exchange rate movements in the six major COVID-19 hot spots—Brazil, China, India, Italy, Turkey, and the United Kingdom. The impact of the COVID-19 deaths on the Rupee/USD, Pound/USD, Yuan/USD, Real/USD, Lira/USD, and Euro/USD exchange rates is analyzed by using the panel ARDL model. The COVID-19 deaths are used as a proxy for market expectations. The panel ARDL model showed a unidirectional long-run causality running from the COVID-19 deaths to the exchange rate. In fact, the coefficient of COVID-19 deaths is positive and significant in explaining the exchange rate(s) in the long run. This result meets the a-priori expectation that a rise in COVID-19 deaths can depreciate the sample countries’ exchange rates. The reason being, the ongoing COVID-19 pandemic has changed the market expectations of the financial market participants about the future value of exchange rate(s) in the major COVID-19 hot spots. Therefore, countries experiencing a sharp daily rise in COVID-19 deaths typically saw their currencies weaken.

## Introduction

To understand the nexus between the COVID-19 crisis and the exchange rate movements in the world’s major economies, one must refer to the subject’s seminal theories. In the long run, the fluctuations in the exchange rates can be attributed to changes in the market fundamentals^1^ or economic variables, such as relative productivity levels, price levels, interest rates, preferences for local or foreign goods, and trade barriers. [30]. However, fluctuations in exchange rates sometimes are too large and impulsive to be explained solely by such factors, for at times, they fluctuate more than 2–3 percentage points within a single day. The variations in the determinants of the exchange rates, in the long run, do not occur recurrently or substantially for explaining exchange rate irascibility. Nevertheless, in the short run, the foreign exchange markets are dominated by transactions in assets such as treasury bills, bank accounts, and stocks. In fact, it has been estimated that approximately 2% of all foreign exchange transactions are related to the exports of goods and services in the short run [7]. The reason being, since most of the reputed international financial markets are strongly connected through sophisticated telecommunications, it allows investors to trade in financial assets on a 24-h basis. Investors can trade rapidly, in various financial assets, in the short run, thereby changing the outlook of currency values almost instantaneously. In the short run, the decisions to hold local or foreign assets are of utmost importance in determining the exchange rates than the exports and imports of goods and services. In this context, in the present unprecedented times of the COVID-19 crisis, the asset market approach for determining short-term exchange rates is highly applicable. Since it rightly argues that in the short run, investors to overcome the conundrum of choice between domestic and foreign assets would consider two critical elements viz. relative real interest rates and expectations about the future exchange rates. Theoretically, the countries in which real interest rates on the financial assets are higher than the rest of the world would expect appreciating currencies^2^ for they would receive substantial amounts of investment funds.^3^ However, expected changes in the interest rates are not the only element taken into consideration by the foreign investors. Expectations about the future path of exchange rates will also guide the investors before denominating their funds in other currencies. At times, in some countries, even if real interest rates are higher than the rest of the world, if one would expect the denominating currency to depreciate considerably, it would receive low amounts of investment funds. Likewise, on occasions, countries receive substantial amounts of investment funds^4^ for one expects their currencies to appreciate in the future. Expectation about the future exchange rates depends upon the market fundamentals and traders’ opinion about the future exchange rates. The impact of the COVID-19 on currency markets worked its way through the channel of changing relative expectations of future economic growth. That is, economies that saw a sharp increase in COVID-19 cases would expect their currencies to depreciate for economic growth expectations would be downgraded in fear that parts of the economy might need to be shut down, thereby reducing the demand for local currency and investment funds therewith. Hence to forestall effort in futility, the present study analyzes the impact of the COVID-19 pandemic on the currency movements of six major COVID-19 hot spots.

### Literature review

In the modern era, the worldwide clampdown of production activities was first witnessed in the aftermath of the world economic depression of 1929 [9, 45] Thereafter, the world economic recession of 2008 also enveloped many parts of the world. The commonality between the two crises was that both resulted from deep financial mismanagement. In contrast, the unprecedented worldwide clampdown measures due to the novel COVID-19 have their genesis in health problems. In early December 2019, the outbreak was caused primarily due to the SARS-COV-2 in Hubei province of China and spread to almost 216 countries in less than a year [38, 49]. The rapid growth of the positively confirmed cases and the subsequent rise of the secondary waves (outbreaks) in the various parts of the world made the situation alarming.^5^ The hasty geographic spread of COVID-19 indicated that the containment of the virus would be challenging for every country globally, i.e., developed or emerging [28]. Subsequently, for its containment, the World Health Organization declared the COVID-19 epidemic a health emergency of international concern on January 31 of 2020 [37, 42]. Later on March 11, 2020, it was declared a pandemic [13, 29]. The COVID-19 pandemic can be considered a major landmark event (economic, social, and political) of the 21 century [50]. The reason is that it resulted in millions of infections and deaths throughout the world. It also stagnated the world economy on a scale not witnessed since the great depression of 1929 [20].

Furthermore, many notable studies [3, 4] have defined COVID-19 as a black swan event^6^, for it consists of all three essential attributes of a black swan event. The reason being, the COVID-19 crisis can be treated as an outlier, for it was beyond the realm of regular expectations, and nothing in retrospect could have convincingly pointed toward its possibility. Besides, it generated repercussions that vehemently challenged economic activity, political stability, and social cohesion throughout the world [31]. The amalgamations of the various types of risks associated with it later cascaded and spread across the global systems in the form of health, economic, and political crises. In this context, infections, such as Bird Flu Asia (2008) and SARS in Hong Kong, were also treated as Black Swan events [19]. Since the COVID-19 crisis had a colossal impact on the global economy, a new term, “Coronomics” was first used by Prof. A. De Alwis [21] for analyzing the negative impact of the novel virus on the economy as a whole. Likewise, a special focus of economists should be directed toward analyzing the impact of the COVID-19 crises on the various crucial sectors of an economy (trade, financial, etc.). In the same vein, [33] rightly argues that it is necessary to acknowledge that the novel COVID-19 crisis had developed a new type of global economic crisis, then a classic type. The negative repercussions are not endogenous but exogenous; these causes are generated outside of the economy and imposed on it by the swift transmission of the COVID-19 infections. Therefore, the present study tried to unravel the economic consequences of COVID-19 by analyzing its impact on the exchange rate of six major COVID-19 hot spots. Due to the *novelty* of the topic, the empirical literature related to the COVID-19 pandemic’s impact on the financial markets in general and exchange rate, in particular, is limited and still developing. Hence, the most relevant and notable studies have been reviewed for better insights into the nexus between the COVID-19 crisis and exchange rate movements.

[28] argued that estimating the prevalence and transmission rate (Contagiousness) of the undocumented COVID-19 cases is critical for comprehending the novel disease’s pandemic potential. The reported cases were documented within china. The findings suggest that almost 86% of all COVID-19 infections were documented before the comprehensive travel restrictions of January 23, 2020. Besides, the transmission rate of undocumented cases per person was recorded as 55%. Moreover, undocumented cases were the source of 79% of the documented cases. The overall findings suggested that the virus’s transmission rate is colossal and would be the biggest stumbling block for all world economies. [47] examined the impact of the COVID-19 outbreak on the emerging stock markets from March 10 to April 30. The empirical results confirmed that the pandemic’s negative impact on the emerging stock markets had gradually declined from mid-April onward.

Moreover, the novel virus’s impact was more robust (high) in the Asian stock markets than in the European emerging markets, wherein the impact was not substantial. The study concluded that the response time and sizes of the fiscal stimulus are prime drivers for offsetting the COVID-19 pandemic effects. [4] employed multifractal detrended fluctuation analysis (MF-DFA) to analyze the efficiency of foreign exchange markets during the initial periods of the COVID-19 outbreak. The overall findings confirmed the presence of Multifractality in the sample FX market. In fact, there was a decline in the efficiency of the foreign exchange markets during the ongoing COVID-19 pandemic. The Australian dollar showed the highest (lowest) efficiency before (during) the COVID-19 crisis assessed in terms of low (high) Multifractality. The study findings would help the policymakers in the said economies design appropriate macroeconomic policies to shape a comprehensive response for improving the efficiency of the foreign exchange markets during the ongoing black swan effect. [27] used the COVID-19 deaths and new confirmed cases to analyze the pandemic’s impact on the USA and China exchange rates. The data on the variables from January 22, 2020, to May 7, 2021, were retrieved from the database of John Hopkins University. The ARDL model verified that the exchange rates of the sample economies were negatively influenced by the two proxies of COVID-19. In this context, the study rightly asserts that a deteriorating currency affects purchasing power, which ultimately cascades into higher inflation. Thus, policymakers in sample economies should analyze the impact of various factors on exchange rates during the present unprecedented times to arrive at robust policy implications. [41] to examine the time-varying pattern caused by the pandemic between exchange rates, temperature, stock returns, and the new COVID-19 infections used the sample of G7 countries. The wavelet coherence and the partial wavelet coherence were employed for empirical analysis. The results inferred that the temperature levels and COVID-19 cases are cyclical, indicating that the former has a material bearing on propagating the COVID-19 infections. Besides, correlation and truncated frequencies revealed that material long-run impact had been observed on exchange rates and stock market returns of sample economies and the COVID-19 cases. In the same vein, [39] added to the literature by employing wavelet and partial wavelet coherence techniques to examine the COVID-19 outbreak effects on exchange rates, stock returns and temperature of the 15 topmost COVID-19-affected countries. The average daily temperature had a significant impact on the spread of the COVID-19 infections in most of the sample economies. Further, the co-movements between COVID-19 cases and exchange rate returns became robust after controlling the stock returns and temperature. [36] studied the information efficiency of the BRICS currency markets during the ongoing COVID-19 pandemic. In the preliminary analysis, the random walk and martingale processes confirmed the evidence of time-varying weak-from market efficiency in the currency markets. The primary empirical modeling used wavelet coherence tools to explore the relationship between COVID-19 deaths and currency returns, and it confirmed that higher frequency components dominate periods of panic and financial turmoil. Moreover, subsequent to the intervention of public authorities in financial markets and the massive roll-out of mass vaccinations, the evidence for the higher frequency oscillations vanished and merely low-frequency co-movements remained. [11] investigated the relationship between Euro/USD exchange rate and oil prices using hourly data from 01/07/2019 to 3/11/2020. With limited evidence, the predictive regression model confirmed that the oil prices significantly influenced the exchange rate. Moreover, the relationship almost disappeared when the effect of COVID-19 outbreak was controlled. The overall results confirmed that COVID-19 affected the Euro/USD exchange rate during March 2020. [24] examined the nature of exchange rate exposure before and during the COVID-19 pandemic. For empirical analysis, the multi-factor arbitrage pricing model and daily data on sectoral and industrial stock returns, market returns, and other economic factors were used. In contrast to sectors, the results verified that the industries were more exposed to exchange rate risk (during) than before the COVID-19 pandemic. Further, results show that exchange rate risk negatively influences most sectors and industries. However, few sectors and industries such as beverages, mining, basic material, and consumer goods remained benefited. Hence, such sectors can be used for diversifying risk during bad times [2]. incorporated TVP-BVAR-SV model to investigate the nature of transmission of quantitative easing on the Euro/USD exchange rate and credit in the EUROZONE during the pre- and post-COVID-19 pandemic. The results confirmed that the responsiveness of Euro/USD exchange rate to the monetary policy shocks substantially varied over time. In fact, quantitative easing did not generate an anticipated effect on the Euro/USD exchange rate during the pandemic; the reason being, the black swan effect has considerably modified investor behavior. [48] used the ARDL procedure to unravel the nexus between oil prices, exchange rates and COVID-19 crises in a sample of five emerging economies. The empirical findings revealed positive co-movements between fatality rates and exchange rates of three of those economies. Besides, oil prices had a negative and significant relationship with the exchange rates of all sample economies. Lastly, oil prices and exchange rate movements impacted the production costs and profitability of the investors. Subsequently, it was rightly recommended that for better insights, the research on currency movements should be broadened against the backdrop of COVID-19 pandemic and oil price crisis that came into being in the first quarter of 2020.

Based on the existing literature on the subject, one can argue albeit the seminal theories on the subject can help us to arrive at appropriate a-priori expectations that COVID-19 deaths can have a robust impact on the foreign exchange market by depreciating the currencies of the major COVID-19 hot spots for the market expectations about the future path of the exchange rates would be downgraded. Nevertheless, since the topic is developing and of global concern, the present study attempts to unravel the nexus between the COVID-19 crisis and exchange rate movements by empirically testing an a-priori hypothesis: the COVID-19 deaths would result in depreciation of the sample economies currencies.

Hence, understanding the relationship between COVID-19 and exchange rate movements is important in assessing macroeconomic performance at the moment. Traders, investors, policymakers, and other stakeholders are all keen to understand the underlying relationship. As a result, we intend to provide evidence on the COVID-19 effects on exchange change rates at the panel level, without limiting our study to a specific country or region. The current study used a large sample from various regions where the impact of COVID-19 on exchange rate movements has garnered little attention to date. In a nutshell, based on the seminal work (Asset-market approach to the determination of exchange rates), this study tested the a priori expectation that an increase in the COVID-19 deaths would depreciate the sample economies’ currencies, causing market expectations about the future path of the exchange rates to be downgraded.

## Methods

The present study employed panel data modeling to analyze the impact of COVID-19 deaths (LTD) on the exchange rate (LEXC)^7^ of six COVID-19 major hot spots, namely Brazil, China, India, Italy, Turkey, and the United Kingdom. The COVID-19 deaths are used as a proxy for market expectations. More precisely, market expectations can be used to forecast the future values of the exchange rates. Any country that is hit hard by the current black swan event would expect its currency to depreciate considerably, for the market expectations would be downgraded in fear that parts of the economy might need to be shut down. Thus, the demand for the domestic currency would decline substantially. The daily data on the COVID-19 deaths and exchange rates of the sample economies^8^ have been retrieved from Our World in Data (COVID-19 Database) and Bank of International Settlements, respectively. The daily observations on the variables have been deliberately used from March 11, 2020,^9^ to December 31, 2020.

Moreover, for robustness, the selection of appropriate estimation procedure was based on the nature of available data. Since our panel consists of a large T (209 days) and small N (6 countries), the panel ARDL framework seemed appropriate and thereby following [15, 16] the said framework was chosen to examine the long-term and short-term cointegration connections between exchange rate and COVID-19 deaths. This framework performs relatively well under circumstances where mutual integration of order zero, I(0), and order one, I(1), exist. The added advantage of the framework is that it gives short-run and long-run estimates simultaneously. ARDL method gives efficient and unbiased estimators to do away with the problems that may arise in the presence of serial correlation and endogeneity [25, 32, 40]. For these reasons, we opt for the ARDL method, a cointegration technique introduced by [35]. The panel ARDL model offers two estimators: the Mean Group Estimator (MG) and the Pooled Mean Group (PMG). [34] suggested the M.G. model fits parameters as averages of the N individual group regressions. For instance, the ARDL model follows

1 $${Y}_{t}= {\alpha }_{i}+ {\gamma }_{i}{{Y}_{i}}_{t-1}+ {\beta }_{i}{X}_{it}+ {\mu }_{it}$$

Here, i stands for country where *i* = 1, 2, 3,…, N and the long-run parameter ${\uptheta }_{i}$ is given by

$${\uptheta }_{i}= \frac{{\beta }_{i}}{1-{\gamma }_{i}}$$

And the M.G. estimator for the whole panel is given by

2 $$\o = \frac{1}{N}\sum_{i=N}^{N}{\uptheta }_{i}$$

3 $$\overline{a} = \frac{1}{N}\sum_{i=N}^{N}{\mathrm{\alpha }}_{i}$$

The above equations illustrate how the model involves estimating a separate regression for each country and calculates the parameters as an un-weighted mean of the individual entities’ estimated coefficients. The M. G. estimator does not impose restrictions on the coefficients to remain the same as it allows all the coefficients among countries to vary. The PMG [2, 4] estimator, on the other hand, involves pooling and averaging of individual regression coefficients. Unlike M.G., PMG constrains the long-run coefficient vector to be equal across panels but allows group-specific short-run and adjustment parameters to differ across groups [5]. The short-run parameters focus on the country-specific heterogeneity, which might be caused by different responses to COVID-19 crises, policy responses to COVID-19, or lockdown strategies. The empirical specification of the PMG model can be specified as

4 $${Y}_{it}=\sum_{j=1}^{p}{\lambda }_{ij}{Y}_{i, t-j}+\sum_{j=0}^{q}{\delta }_{ij}{X}_{i, t-j}+{\epsilon }_{t}+{\mu }_{it}$$

where i is the number of cross sections (i = 1, 2, 3,…, N) and time t = 1, 2, 3,…,T. ${X}_{it}$ is a vector of K * 1 regressors, ${\epsilon }_{t}$ is a group-specific effect, and $\lambda ij$ is a scalar. If the variables follow I(1) order and are cointegrated, then the disturbance term is an I(0) process. The main feature of cointegrated variables is their convergence to any deviation from long-run equilibrium. This feature infers from error correction dynamics of the variables in the system, and therefore, it is common to re-parameterize the above equation into an error correction system as

5 $$\Delta {Y}_{it}= {\Phi }_{i}{Y}_{i,t-j}-{\theta }_{i}{X}_{i,t-j}\sum_{j=1}^{p-1}{\lambda }_{ij}{\Delta Y}_{i, t-j}+\sum_{j=0}^{q-1}{\delta }_{ij}{\Delta X}_{i, t-j}{\epsilon }_{t}+{\mu }_{it}$$

The error correction term ${\Phi }_{i}$ shows the speed of adjustment toward long-run equilibrium.

A choice between M.G. and PMG is based on the [18] which says that once the P value is greater than 5%, then PMG estimations will be preferred.

### Panel unit root test (PURT)

For efficient and consistent estimation of the cointegration, relationship’s ARDL model requires that the variables under study must be either integrated of order I(0) or integrated of order I(1) or must be mutually integrated. However, none should be integrated of order I(2)—estimations are not consistent when some variables are integrated of order two. Therefore, we employed the LCC test and IPS test to ensure this crucial assumption of the ARDL cointegration. The LLC [26] test presumes a common unit-roots process so that the autoregressive coefficients are homogenous across countries [43]. The general form of LLC test is specified as

6 $$\Delta {Y}_{i,t}= {\gamma }_{0i}+ {{\rho }_{i}Y}_{it-1}+ \sum_{i=1}^{Pi}{\gamma }_{1i}{\Delta Y}_{i,t-j}+{\mu }_{i,t}$$

In Eq. (6), ${\gamma }_{0i}$ is a constant term which is supposed to vary across the countries, ρ is the homogenous autoregressive coefficient, P_i_ denotes the lag order and ${\mu }_{i,t}$ is an error term, which is supposed to be independent across individual entities and follows ARMA stationary process for every cross section.

7 $${\mu }_{i,t}=\sum_{j=0}^{\infty }{\gamma }_{1i}{\Delta Y}_{i,t-j}+ {\varepsilon }_{i,t}$$

The null and alternative hypotheses of the test are as follows:

$${H}_{0}= {\rho }_{i}=0 for all i$$

$${H}_{1}={\rho }_{i}<0 for all i$$

The IPS test [22], contrary to the LLC test, allows for individual unit root processes and heterogeneous autoregressive coefficients across entities. A series may be represented by ADF as follows:

8 $$\Delta Y_{{i,t}} = \mathop W\limits^{'} _{i} + \rho _{i} Y_{{it - 1}} + \sum\limits_{{j = 1}}^{{Pi}} {\rho _{{ij}} } \Delta Y_{{i,t - j}} + V_{{i,t}}$$

IPS test allows for heterogeneity in ${V}_{i}$.The IPS test provides a separate unit root test for every cross-sectional unit. If ${t}_{iT}$ is an individual t-statistic for every i, then the IPS unit root test is based on the average of all individual statistics of the ADF test defined as

9 $$\mathop t\limits^{'} _{T} = \frac{1}{N}\sum\limits_{{i = 1}}^{N} {t_{{iT}} }$$

where ${t}_{iT}$ test statistic is calculated as follows:

10 $$\mathop A\limits^{'} _{t} = \frac{{\sqrt {\text{N}} \left[ {{\text{t}}_{{\text{T}}} - {\text{E}}({\text{t}}_{{\text{T}}} )} \right]}}{{\sqrt {{\text{Var}}({\text{t}}_{{\text{T}}} )} }}$$

### Dumitrescu and Hurlin Panel Causality Test

In this paper, we employed the panel causality test introduced by [14]. This test is a simple version of [17] non-causality test for heterogeneous panel data models with fixed coefficients. This test considers two dimensions of heterogeneity; the heterogeneity of the causal relationships and the heterogeneity of the regression model used to test the Granger causality. The underlying regression model for the D.H. panel causality test is specified as

11 $${Y}_{i,t}={\delta }_{i}+\sum_{j=1}^{k}{\alpha }_{1j}{Y}_{i,t-i}+\sum_{j=1}^{k}{\beta }_{1j}{X}_{i,t-i}+{\varepsilon }_{i,t} i=1, 2, 3,\dots ,N and t=\mathrm{1,2},3,\dots ,T$$

where k is lag order, which is assumed to be identical for all i,${Y}_{i,t}$ and ${X}_{i,t}$ are two stationary variables for country i at time t and ${\delta }_{i}$ signifies time-invariant individual effects. The autoregressive parameters ${\alpha }_{1j}$ and regression slope coefficients ${\beta }_{1j}$ are allowed to vary across entities. ${Y}_{i,t}$, and ${X}_{i,t}$ are interchanged in Eq. (11) to test for causality.

Under the null hypothesis, we assume that there is no causality relationship for any of the panel units. This assumption is called the homogeneous non-causality hypothesis, which is specified as

$${H}_{0}: ={\beta }_{1j}\dots = {\beta }_{1k}=0 {\forall }_{i}=1,\dots , N$$

This corresponds to the absence of causality for all individuals in the panel. The test assumes that there can be the causality for some entities but not necessary for all. The alternative hypothesis, therefore, is defined as

$${H}_{0}: ={\beta }_{1j}\dots = {\beta }_{1k}=0 {\forall }_{i}=1,\dots , N$$

$${\beta }_{1j}\ne 0 or\dots or {\beta }_{1k}\ne 0 {\forall }_{i}={N}_{1}+1,\dots , N$$

where ${N}_{1}\in [0, N-1]$ is unknown. If ${N}_{1}=0$, there is causality for all entities included in the panel. ${N}_{1}$ must be strictly lower than N; otherwise, there is no causality for all entities, and H_1_ reduces to H_0._

Against this background, Dumitrescu and Hurlin suggested the following three-step procedure to obtain the average Wald statistic;(a) estimate the regression equation enclosed in (11) for all countries; (b) perform F-tests of the K linear hypotheses ${H}_{0}: ={\beta }_{1j}\dots = {\beta }_{1k}=0$ to retrieve $W$, and (c) finally compute the average of individual-country Wald statistics. The average Wald statistic $\overline{W }$ is

12 $$\overline{W }=\frac{1}{N}\sum_{I=1}^{N}Wi$$

where $Wi$ is the standard adjusted Wald test statistic for individual country i observed during T periods. It is to be noted that the test is designed to detect causality at the panel-level, and rejecting H_0_ does not exclude that there is no causality for some individuals. Under the assumption that $Wi$ is identically and normally distributed, it can be shown that the standardized statistic $\overline{Z }$ when T → ∞ first and then N → ∞, that is T > N panel, follows a standard normal distribution;

13 $$\overline{Z }=\sqrt{\frac{N}{2K}} .\left(\overline{W }-K\right)\to N\left(\mathrm{0,1}\right)$$

Also, for a fixed T dimension with T > 5 + 3 K and N → ∞, that is N > T panel, the approximated standardized statistic $\tilde{Z }$ follows a standard normal distribution:

14 $$\tilde{Z} = \sqrt {\frac{N}{{2K}}.\frac{{T - 3K - 5}}{{T - 2K - 3}}} .\left[ {\frac{{T - 3K - 3}}{{T - 3K - 1}}.\overline{W} - K} \right] \to N\left( {0,1} \right)$$

The testing procedure of the null hypothesis in (14) is finally based on $\overline{Z }$ and $\tilde{Z }$. If these are larger than the corresponding critical values, then one should reject the null hypothesis and conclude that there is Granger causality among variables under study.

## Results and discussion

### Descriptive statistics

Table 1 reports descriptive statistics of the variables under study. It is to be noted here that all the variables were subjected to natural logarithms to decrease the data variability. It can be seen from Table 1 that the exchange rate of the sample countries varied from 4.34 to − 0.31 with a mean value of 1.59 and a standard deviation of 1.53. Similarly, sample countries’ magnitude of COVID-19 deaths varied from 0.00 to 11.92, with a mean value of 9.16 and a standard deviation of 2.02.

**Table 1:** Descriptive statistics

Variable/Statistic	LEXC	LTD
Mean	1.585854	9.156402
Median	1.801391	9.423749
Maximum	4.342979	11.91971
Minimum	− 0.311093	0.00000
Std. dev.	1.531751	2.020935
Skewness	0.422153	− 1.716510
Kurtosis	2.291344	7.181938
Sum	1979.146	11,427.19
Sum std. dev.	2925.789	5092.971
Observations	1248	1248

The cursory view of the data in Table 1 shows that the exchange rate of sample countries has positive mean values; their exchange rates have depreciated over the period. However, it is difficult to draw conclusions about the nature of the association between COVID-19 deaths and exchange rates based on this brief examination of the data, prompting the necessity for an empirical investigation.

### Results of panel unit root test(s)

The PURT summarized in Table 2 shows that interest variables have both non-stationary and stationary characteristics. LEXC is I(1) according to LLC and IPS, while LTD is I(0) according to IPS and I(1) as per the LLC test. Since none of the variables is of order I(2), the ARDL cointegration approach’s crucial assumption is fulfilled. Therefore, panel ARDL appears to be the most appropriate estimation procedure for the present study (Table 3).

**Table 2:** Results of PURT

Series	Method	Statistic	Prob.	Cross sections	Obs.
*Results of LLC test*					
LEXC	LLC	0.61748	0.7315	6	1242
D (LEXC)	LLC	− 42.4890	0.00000***	6	1242
LTD	LLC	6.36	1.0000	6	1200
D(LTD)	LLC	− 4.04	0.000***	6	1194
*Results of IPS test*					
LEXC	IPS	0.37801	0.6473	6	1242
D (LEXC)	IPS	− 38.0998	0.0000***	6	1242
LTD	IPS	− 3.76445	0.0001***	6	1242

^***^Level of significance at 1%

**Table 3:** Hausman test results

Test summary	Chi-sq. statistic	Chi-sq. d.f	Prob.
Cross section random	4.12	1	0.1272

### Mean group and pooled mean group models

Can the impact of COVID deaths daunt the long-run exchange rate? Table 4 reports the estimation results of pooled mean group model. The PMG model results provide the short-run and long-run impacts of COVID-19 deaths on currency movements (appreciation or depreciation). The estimated results’ direction focuses more on PMG as the Hausman test confirmed it is efficient and suitable than M.G.^10^ (Table 3).

**Table 4:** Pooled mean group estimates

Regressor	Coef	Std. Err.	t-Statistic	Prob.
Dependent variable; Exchange rate				
*Long-run estimates*				
LTD	0.062597	0.013186	4.747203	0.0000***
*Short-run estimates*				
ECT	− 0.048412	0.019474	− 2.485950	0.0131**
D (LEXC)_t-1_	− 0.074056	0.034461	− 2.185950	0.0318**
D(LEXC)_t-2_	0.062699	0.034584	1.812914	0.0701*
D(LTD)	0.032908	0.018303	1.977966	0.0724*
D(LTD)_t-1_	0.0044235	0.012178	0.347766	0.7281
Trend	− 2.85E-05	1.34E-05	− 2.123325	0.0339**
Cons	0.026885	0.036575	0.735071	0.4624

^*^, **, and *** represent 10, 5 and 1% level of significances, respectively

The PMG long-run estimators show that total COVID-19 deaths (LTD) positively and significantly impact the sample economies’ exchange rates. This result meets the a-priori expectation that a rise in COVID-19 deaths can depreciate the sample countries’ exchange rates. Therefore, countries seeing sharp daily rises in COVID- 19 deaths typically saw their currencies weaken [10, 12, 44, 51, 56]. The possible reason for this result might be that these economies are/were the epicenters of the COVID-19 crises, and this new epidemic, akin to previous global disasters, has produced significant foreign exchange movements in these epicenters. However, the dynamics of foreign exchange movements has been noticeably faster in the current COVID-19 crisis. At the same time, compared to prior crises, the volume of capital outflows from emerging economies has been significantly higher [53]. Although the impact of the COVID-19 deaths with one year lag on the exchange rate in the short run is positive, it is insignificant. The results also show that today’s exchange rate is positively and significantly influenced by current day’s COVID-19 deaths in the short run. Therefore, the exchange rates and the COVID-19 deaths show discernible co-movements, although the magnitude of their co-movement differed across regions [37, 46]. Further, the two periods lagged exchange rate coefficient with a positive sign might represent an additional risk factor to investors in foreign exchange markets. Given the study’s emphasis, such findings should be considered carefully.

The error correction term (ECT) is negative and statistically significant as anticipated [1]. This shows that the exchange rate adjusts toward its long-run equilibrium path at the rate of 4.84% each day. Further, it implies a unidirectional long-run relationship/causality running from COVID-19 deaths to the sampled countries’ exchange rate [8].

### Short-run estimates of major six COVID-19 hot spots

The short-run parameters that focus on the country-specific heterogeneity are reported in Tables 5, 6, 7, 8, 9, 10

**Table 5:** Short-run estimates based on PMG for India

Regressor	Coef	Std. Err.	t-statistic	Prob.|
*Dependent variable; Exchange rate*				
ECT	− 0.006866	2.24E-05	− 306.5230	0.0000***
D (LEXC)_t-1_	− 0.219259	0.004939	− 44.39123	0.0000***
D(LEXC)_t-2_	− 0.030092	0.0044758	− 6.324174	0.0000***
D(LTD)	0.0033909	1.43E-05	237.1258	0.0000***
D(LTD)_t-1_	0.003909	1.19E-05	328.6798	0.0000***
Trend	1.47E-05	1.47E-10	100,405.2	0.0000***
Cons	− 0.027569	0.000363	− 75.95988	0.0000***

^***^represents 1% level of significance

**Table 6:** Short-run estimates based on PMG for the United Kingdom

Regressor	Coef	Std. Err	t-Statistic	Prob.|
*Dependent variable; Exchange rate*				
ECT	− 0.053262	0.000235	− 226.8807	0.0000***
D (LEXC)_t-1_	− 0.006628	0.004373	− 1.515481	0.2269
D(LEXC)_t-2_	0.103389	0.004356	23.73475	0.0002***
D(LTD)	0.030027	7.39E-05	406.4076	0.0000***
D(LTD)_t-1_	0.027618	6.26E-05	440.8620	0.0000***
Trend	− 2.57E-05	1.85E-05	− 138,933.7	0.0000***
Cons	− 0.046517	0.000124	− 374.9914	0.0000***

^***^Represents 1% level of significance

**Table 7:** Short-run estimates based on PMG for China

Regressor	Coef	Std. Err	t-Statistic	Prob.
Dependent variable; Exchange rate				
ECT	− 0.129473	0.000876	− 147.8105	0.0000***
D (LEXC)_t-1_	− 0.133257	0.004527	− 29.43905	0.0001***
D(LEXC)_t-2_	0.004903	0.004090	1.198849	0.3166
D(LTD)	0.008798	5.75E-05	152.9587	0.0000***
D(LTD)_t-1_	− 0.006742	5.73E-05	− 117.6824	0.0000***
Trend	− 7.92E-05	3.29E-10	− 240,992.6	0.0000***
Cons	0.189731	0.001653	114.7837	0.0000***

^***^Represents 1% level of significance

**Table 8:** Short-run estimates based on PMG for Brazil

Regressor	Coef	Std. Err	t-Statistic	Prob.
*Dependent variable; Exchange rate*				
ECT	− 0.061035	0.000374	− 163.1563	0.0000***
D (LEXC)_t-1_	− 0.016083	0.004356	− 3.691642	0.0345**
D(LEXC)_t-2_	0.105758	0.004319	24.48004	0.0001***
D(LTD)	0.054543	0.000868	62.84917	0.0000***
D(LTD)_t-1_	0.036657	0.000574	63.83433	0.0000***
Trend	− 4.98E-05	9.38E-10	− 53,073.49	0.0000***
Cons	0.064967	0.000642	101.2345	0.0000***

^**^, ***Represents 5% and 1% level of significances, respectively

**Table 9:** Short-run estimates based on PMG for Turkey

Regressor	Coef	Std. Err	t-Statistic	Prob.
*Dependent variable; Exchange rate*				
ECT	− 0.009002	0.000211	− 42.71297	0.0000***
D (LEXC)_t-1_	− 0.032242	0.004929	− 6.541744	0.0345**
D(LEXC)_t-2_	0.191498	0.004822	39.71447	0.0001***
D(LTD)	− 0.004999	7.17E-05	− 69.77593	0.0000***
D(LTD)_t-1_	0.011487	5.33E-05	215.4665	0.0000***
Trend	− 5.82E-06	2.15E-10	− 26,974.36	0.0000***
Cons	0.014235	0.000416	34.25370	0.0000***

^**^, ***Represents 5% and 1% level of significances, respectively

**Table 10:** Short-run estimates based on PMG for Italy

Regressor	Coef	Std. Err.	t-Statistic	Prob.
*Dependent variable; Exchange rate*				
ECT	− 0.044567	0.000499	− 98.33049	0.0000***
D (LEXC)_t-1_	− 0.036869	0.004857	− 7.590988	0.0047***
D(LEXC)_t-2_	0.000767	0.004647	0.164988	0.8794
D(LTD)	0.112068-	0.000973	115.2097	0.0000***
D(LTD)_t-1_	− 0.047516	0.000891	− 53.33798	0.0000***
Trend	2.55E-05	2.69E-10	94,553.49	0.0000***
Cons	− 0.033554	0.000290	− 115.8252	0.0000***

^***^Represents 1% level of significance

The short-run estimates of the selected Panel ARDL (3, 2) confirm that in all COVID-19 major hot spots, the COVID-19 deaths and the exchange rates are positively related. This implies that an increase in the COVID-19 deaths depreciates each sample economy’s domestic currencies, albeit the magnitudes differ across the countries. This might be caused by different responses to COVID-19 crises, policy responses to COVID-19, or lockdown strategies [52, 54, 55]. The finding seems to be robust in the long run as well, for, in each sample economy, the error correction term is negative and significant.

### Results of dumitrescu and hurlin panel causality test

The D.H. panel causality test results are presented in Table 11. The results reveal that COVID-19 deaths homogeneously cause exchange rate, but exchange rate does not homogenously cause COVID-19 deaths [6]. These results again suggest that there is a homogenous unidirectional causality running from LTD to LEXC. The causal association between exchange rate and COVID-19 fatalities could be due to the meltdown of financial markets during the lockdowns [38]. Moreover, most of the disturbances^11^ during the crisis can be attributed to the channel running from COVID-19 deaths to exchange rates. In a nutshell, one can use COVID deaths to predict better the exchange rate movements than simply by using the past pattern of exchange rates. This result can be interpreted as movements in the COVID-19 fatalities that appear to lead to the exchange rate in the case of sampled COVID-19 hot spots.

**Table 11:** Results of panel causality test

Null hypothesis	W-stat	Zabar-Stat	Prob
LTD does not homogenously cause LEXC	4.75204	3.27906	0.0010***
LEXC does not homogenously cause LTD	3.32031	1.56053	0.1136

^***^Level of significance at 1%

## Limitation and future research

The current paper has enormous future potential. Future research could, for example, use data to expand the sample to include more hard-hit COVID-19 countries, such as Mexico, Russia, France, Indonesia, and others, to further investigate the impact of COVID-19 on the dynamics of exchange rates. Furthermore, because the third wave of the recent COVID-19 pandemic is still ongoing, more research with a larger dataset to account for the third and subsequent waves would be beneficial. Besides, other relevant variables, such as the number of successful vaccinations, interest rates, trade flows, and investment flows, could be incorporated into the model. Since, significant trading activity in the world takes place in USD; the nominal exchange rates of domestic currencies vis-à-vis USD were used as the proxy variables to measure their respective exchange rate movements. However, for deeper insights, the exchange rate movements could be measured using other dominant currencies- British pound sterling, Euro, etc.

## Conclusion

The study’s primary objective was to unravel the nexus between the COVID-19 crisis and the exchange rate movements in the six major COVID-19 hot spots. More precisely, the impact of the COVID-19 deaths on the Rupee/USD, Pound/USD, Yuan/USD, Real/USD, Lira/USD, and Euro/USD exchange rates was analyzed from March 11, 2020, to December 31, 2020. The COVID-19 deaths were used as a proxy for market expectations. Since our panel consisted of a large T (209 days) and small N (6 countries), the panel ARDL model was considered to be an appropriate technique for examining the long-term and short-term cointegration connections between exchange rate and COVID-19 deaths. The panel ARDL model offers two estimators: the Mean Group Estimator (MG) and the Pooled Mean Group (PMG). A choice between MG and PMG was based on the Hausman test, and the same verified that PMG is appropriate than MG. The PMG long-run estimators showed that total COVID-19 deaths positively and significantly impacted the exchange rate. Further, the Dumitrescu and Hurlin panel causality test results showed that COVID-19 deaths homogenously cause exchange rates in the sample economies. Based on empirical findings, the sample economies’ policymakers should devise and restructure macroeconomic and COVID-19 response policies for improving the efficiency of foreign exchange markets during the present unprecedented times. Hence, to kickoff the economic recovery plans, its incumbent upon the governments of respective economies to strengthen monetary, fiscal, and financial policy interventions.

## Data Availability

Yes.

## Abbreviations

ARDL: Auto regressive distributed lag model
COVID-19: Corona virus disease
PMG: Pooled mean group
PURT: Panel unit root test
TGARCH: Thresh-hold generalized auto regressive conditional heteroscedasticity

## Footnotes

1 This framework for determining the exchange rates is known as Balance of Payments approach. It analyzes the flow of goods and services, investment funds and their impact on foreign exchange rates/transactions.

2 Demand for the local currencies would increase substantially.

3 Conversely holding other things constant, countries having low levels of real interest rate would expect depreciating currencies.

4 Albeit they have lower real interest rates than the rest of the world.

5 Due to the high transmission rate of the disease and its global consequences, it is being compared to the influenza pandemic of 1918.

6 Depending upon the individual interpretations’ of such event, many studies contradict that COVID-19 is not a black swan.

7 The L in the prefix of the selected variables indicates that the variables have been log-transformed.

8 Domestic daily exchange rates vis-à-vis USD is Rupee/USD, Pound/USD, Yuan/USD, Real/USD, Lira/USD, and Euro/USD.

9 On March 11 2020, COVID-19 was declared as a pandemic.

10 For brevity PMG model has been reported and interpreted only.

11 Note: The disturbances are in the form of reduction in production activities, trade flows, investments flows which substantially explain the movement of exchange rates.
